# Sex Differences in the Associations Among Parenting, Socioeconomic Status, and Error Monitoring Among Adolescents

**DOI:** 10.1002/dev.70023

**Published:** 2025-02-11

**Authors:** Saad Pirzada, Emilio A. Valadez

**Affiliations:** ^1^ Department of Human Development and Quantitative Methodology University of Maryland College Park Maryland USA; ^2^ Department of Psychology University of Southern California Los Angeles California USA

**Keywords:** EEG, error‐monitoring, parenting, sex differences, socioeconomic status

## Abstract

The error‐related negativity (ERN) is a frontocentral deflection in the human EEG that is sensitive to error commission. Past research indicates that the ERN is modulated by individual differences in socioeconomic status (SES) and parenting style; however, there is limited research examining sex‐differences in how these factors influence the ERN. The present study aimed to elucidate the relations among SES, parenting style, sex, and the ERN. In this study, 176 participants from a relatively large longitudinal study performed a Flanker task at age 15 years to measure the ERN. At the same assessment time, parenting style was assessed via parent report using the Parenting Styles and Dimension Questionnaire (PSDQ). Parents reported on their highest level of education which was used as an indicator of household SES. Authoritarian and permissive parenting scores each significantly moderated the relation between maternal education and ERN amplitudes, but in both cases this moderation differed by child sex. There were no significant direct associations between maternal education and ERN amplitude or between parenting scores and ERN amplitude. Overall, findings may suggest sex differences in the impact of social context on error monitoring development. This study highlights (1) that parenting behaviors may modulate the impact of SES on cognitive control and and (2) the importance of considering sex differences when examining the interplay between SES, parenting, and cognitive control.

Effective adaptation to dynamic surroundings relies on careful monitoring of one's errors and behaviors (Falkenstein et al. 2000; Botvinick et al. 2001; Holroyd and Coles 2002; van Veen and Carter 2002). The error‐related negativity (ERN) is a negative voltage deflection measured via electroencephalography (EEG) sensitive to error commission and thought to reflect error monitoring processes (Falkenstein et al. 1991; Gehring et al. 1993; Hajcak 2012). The ERN is maximal among frontocentral electrode sites (Meyer 2012). Conceptually, errors can be thought of as motivationally salient internal conflicts which pose a danger or threaten safety and require a prompt response (Meyer and Wissemann 2020). Accordingly, larger or more costly errors are associated with larger amplitude ERN signals (Hajcak et al. 2005; Chiu and Deldin 2007; Ganushchak and Schiller 2008; Endrass et al. 2010). Moreover, perception of errors influences the ERN, in that the ERN may serve as an index of the mismatch between an individual's expected performance and their actual performance (Perrone‐McGovern et al. 2017). Punishment, too, can modulate the salience of errors, such that previous studies of college‐aged students have found that errors punished with a loud noise or shock induce increased ERNs even in subsequent errors after the punishments have terminated (Riesel et al. 2012; Meyer and Gawlowska 2017). Additionally, adolescents who have experienced more trauma (Lackner et al. 2018) and veterans that have greater exposure to battle (Khan et al. 2018) both exhibit enhanced ERNs. Prior work suggests that these effects are explained by stress and may be cumulative such that adolescents who have experienced more cumulative stress have enhanced ERNs (Banica et al. 2021). It could be that situations perceived to be more dangerous necessitate greater error monitoring, as seen by the downstream mobilization of defense systems following errors (Weinberg et al. 2011).

Given that behavioral contingencies appear to modulate the ERN, it may be unsurprising that several contextual factors have been shown to relate to ERN amplitude. Certain parenting styles, including punitive and controlling styles which more frequently and severely punish children for their errors (Robinson et al. 2001), are associated with heightened concern about errors and with larger ERNs among children (Banica, Sandre, and Weinberg 2019; Kawamura, Frost, and Harmatz 2002; Meyer and Wissemann 2020). Socioeconomic status (SES) is another factor that broadly reflects other contextual factors in development including stress and hardships (Bradley and Corwyn 2002). Moreover, parental education level is linked to stress perception in adolescents such that adolescents from households with lower parental education levels have higher perceived stress than adolescents from households with higher parental education levels (Finkelstein et al. 2007). Although the literature on the direct relation between SES and ERN is scant, a few studies suggest that low‐SES in children is associated with smaller ERNs; additionally, in children higher SES is associated with larger ERNs (Perera‐W et al. 2021; Chong, Mirzadegan, and Meyer 2020). Another study found no significant difference in ERN between low SES and high SES groups; however, it found that preschoolers who experience high maternal sensitivity from higher SES homes show an increase in ERN over time (Brooker 2018). Previous studies also suggest that parenting behaviors can buffer the effects of low income on executive functioning more generally. Specifically, high paternal discipline buffers the detrimental effects of income on executive functioning in adults who grew up in low‐income homes (Liu and Lachman 2019).

Both low SES (Reiss et al. 2019) and less responsive and less stimulating parenting (Marsh, Dobson, and Maddison 2020) contribute to greater stress exposure among early adolescents. However, there are also sex differences in sensitivity to environmental stressors, with heightened sensitivity in females, specifically stress‐induced hyperarousal (Bangasser et al. 2018). Moreover, specific parenting styles, derived from Baumrind's three models of parental control, including permissive, authoritarian, and authoritative (Baumrind 1966), characterized by varying levels of responsiveness and demands of parents on children, have differential effects on stress perception among males and females. Permissive parenting, characterized by high responsiveness and low demands, is positively associated with increased stress perception in females but not males when exhibited by mothers (Barton and Kirtley 2012); on the other hand, authoritarian parenting, characterized by low responsiveness and high demands, was not associated with stress perception in females, but paternal authoritarian parenting was positively associated with stress perception in males. Additionally, the only parenting style not associated with the highest stress level in either males or female adolescents is authoritative parenting, characterized by high responsiveness and high demands (Ramesh et al. 2022). Previous studies find that the relations between the ERN and individual difference measures vary based on sex. So far, such sex differences have been particularly evident in the ERNs links to anxiety. Meta‐analytic findings based on 37 separate samples suggest that the relation between the symptoms of anxiety and the ERN is significantly stronger in females than males (Moser et al. 2016), underscoring the need to explore sex differences in error‐monitoring research and suggesting possible sex differences in error‐monitoring‐related behavior. Although prior research has found enhanced ERNs in adolescents and adults following early childhood stress exposure (Mehra, Hajcak, and Meyer 2022) and adversity (Wu et al. 2019), these studies do not examine the impact of sex. Additionally, prior research has suggested enhanced ERNs in females who receive greater authoritarian parenting (Banica, Sandre, and Weinberg 2019) and no change in ERN in those receiving authoritative and permissive parenting (Chong, Mirzadegan, and Meyer 2020); however, these studies too do not take into account sex and socioeconomic status, raising the need to explore possible sex and socioeconomic differences in the links between stress exposure, parenting, and the ERN.

Given that adolescence appears to be a sensitive period during which contextual factors may have an outsized effect on child behavior relative to some other stages of development (Fuhrmann, Knoll, and Blakemore 2015), we predict that in adolescents the combined effects of adverse parenting and SES conditions together lead to enhanced ERNs, with potential sex differences. Specifically, because past work finds that female adolescents show greater biological reactivity to psychosocial stress (Ordaz and Luna 2012), we hypothesize that both authoritarian and permissive parenting when combined with low SES leads to enhanced ERNs in females relative to the smaller changes in males. The present study addresses this question using electrophysiological and parent‐reported data from 176 adolescents enrolled in a relatively large longitudinal study.

## 1 Methods

### 1.1 Participants

Participants were drawn from a larger longitudinal study (*N* = 291) examining relations among infant temperament and anxiety (Hane et al. 2008). Briefly, infants (*N* = 779; age 4 months) were recruited from the greater Washington D.C. metropolitan area and completed a laboratory temperament screening for emotional and motor reactivity towards novel auditory and visual stimuli. From these, infants with high motor and high positive or high negative reactivity were oversampled to reflect a range of temperamental reactivity that is wider than would be found in a randomly selected community sample. The selected infants (*N* = 291) continued to participate in assessments of cognitive and socio‐emotional development throughout childhood and adolescence. Of these, 176 participants had data for our main variables of interest and were included in the present analyses. At the time of the EEG assessment, youth were approximately 15 years old (mean = 15.4 years, SD = 0.6 years). Informed consent and assent were obtained at each assessment, and each visit protocol was approved by the institutional review board of the University of Maryland.

### 1.2 Socioeconomic Status (SES)

Upon enrollment in the longitudinal study, participants’ mothers reported on their highest level of education, which was used as a proxy for SES. We focused on this early childhood measure of SES because this was thought to be the best representation of SES throughout childhood, in light of findings that SES during early childhood tends to be stable over time (Zhang, Liu, and Choi 2020). Maternal education was coded on an ordinal scale where high school diploma = 0, college degree = 1, and postgraduate degree = 2. Therefore, higher scores indicate greater educational attainment. For descriptive statistics and bivariate correlations among key study variables (see Table 1).

**TABLE 1:** Descriptive statistics and bivariate correlations among key study variables.

Measure	*N*	Mean	SD	1	2	3	4	5	6	7	8
1. Maternal education	273	1.21	0.72								
2. PSDQ Authoritative	176	106.96	13.73	0.00							
3. PSDQ Authoritarian	176	37.07	7.83	−0.16*	−0.10						
4. PSDQ Permissive	176	33.13	5.51	0.10	0.10	0.51***					
5. ERN amplitude (microvolts)	125	−2.84	2.18	0.07	−0.04	0.08	0.15				
6. CRN amplitude (microvolts)	125	−0.64	1.52	0.08	−0.11	−0.01	0.1	0.12			
7. Pubertal developmental scale	170	2.53	0.50	−0.05	0.06	−0.21*	−0.16	−0.01	−0.03		
8. Sex (% male)	291	46.39%	—	0.01	0.01	0.13	0.04	−0.06	0.03	−0.52***	
9. Race/ethnicity (% White)	290	69.66%	—	0.19**	0.16*	−0.20**	0.09	0.19*	−0.04	−0.06	−0.05

*Note:* Reported *N*s reflect the number of participants with valid scores for the given measure. Sex was dummy coded as female = 0 and male = 1. Maternal education was coded on an ordinal scale where high school diploma = 0, college degree = 1, and postgraduate degree = 2. Lastly, due to small cell sizes for specific racial/ethnic groups, race/ethnicity was collapsed and dummy coded as non‐White = 0 and White = 1.

PSDQ = Parenting Styles Dimension Questionnaire; ERN = error‐related negativity; CRN = correct‐related negativity.

^*^ *p* < 0.05.

^**^ *p* < 0.01.

^***^ *p* < 0.001.

### 1.3 Questionnaires

At age 12 years the Pubertal Development Scale (PDS; Petersen et al. 1988) was administered to participants which consists of a section of self‐report measures that involves puberty as well as a section that is about physical development, including development status of features such as pubic hair, height, and skin changes for both girls and boys as well as facial hair growth and voice change in boys. The purpose of the PDS is to assess pubertal status.

At age 15 years the Parenting styles and dimension questionnaire (PSDQ) was administered to parents (Robinson et al. 1995). The PSDQ is a 62‐item questionnaire that asks caregivers to report on their own parenting behaviors. It includes three subscales, each reflecting Baumrind's (1971) authoritative, authoritarian, and permissive typologies. For each subscale, higher scores indicate greater use of that parenting style. The authoritative and authoritarian subscales each showed good internal consistency (Cronbach's alpha = 0.89 and 0.83, respectively), but the permissive subscale had lower internal consistency, likely due to the smaller number of items (Cronbach's alpha = 0.57). Specifically, the authoritative and authoritarian subscales had 27 and 20 items, respectively, whereas the permissive subscale had 15 items.

### 1.4 Flanker Task

At the age of 15, participants completed a flanker task while their brain activity was recorded via EEG. Participants sat approximately 70 cm away from a 15‐inch computer monitor which displayed congruent (e.g., < < < < <) or incongruent (e.g., < < > < <) sets of arrows. Specifically, participants were asked to identify the directionality of the middle arrow in the set of arrows regardless of the directionality of the arrows other than the middle arrow. Each trial began with a cue that resembled an asterisk in the middle of the screen for 200 ms, followed by a screen that was blank, and lastly the target display for 250 ms. Participants had to respond within a frame of 1100 ms. As a practice session prior to beginning the task, participants completed a block of 20 trials in order to become familiar with the computer stimuli and button box. After the practice session, participants completed a total of 320 trials, consisting of 10 blocks of 32 trials. Within each block, congruent trials and incongruent trials were presented with equal probability. After each block, computer‐generated feedback was shown to the participants to ensure a sufficient error rate for ERN measurement (Buzzell et al. 2017). Specifically, if accuracy during the block was at or below 75%, the feedback would tell participants to “be more accurate.” If block accuracy was at or above 90%, participants would be told to “respond faster.” Lastly, if accuracy was between 75% and 90%, participants would be told that they were doing a “good job”.

### 1.5 EEG Recording and Preprocessing

During the flanker task, electroencephalograph (EEG) data was acquired using a 128‐channel HydroCel Geodesic Sensor Net and EGI software (Electrical Geodesic, Inc., Eugene, OR). Electrode impedances were maintained below 50 kΩ. Data were sampled at 250 Hz, and referenced online to electrode Cz. All pre‐processing, including ocular artifact detection and removal, was performed using the Maryland analysis of developmental EEG (MADE; Debnath et al. 2020) pipeline, which utilizes MATLAB (The MathWorks, Natick, MA) functions from EEGLAB (Delorme and Makeig 2004) and its plugins “FASTER” (Nolan, Whelan, and Reilly 2010) for bad channel removal, and “ADJUST” (Mognon et al. 2011) and “ADJUSTED ADJUST” (Leach et al. 2020) for automated removal of ICA components containing ocular artifacts. Specific parameters included applying a high‐pass filter at 0.3 Hz and low‐pass filter at 50 Hz. Incongruent error and incongruent correct epochs were extracted from –100 to 500 ms with respect to the button response. Baseline correction was applied by subtracting the average voltage during a –50 to 0 ms baseline period from each trial's voltage values. Following baseline correction, epochs were excluded on a per‐channel basis if they contained voltage values exceeding a threshold of ±125 microvolts and any epochs in which more than 10% of nonocular channels exceeded this threshold were marked bad; otherwise, bad channels were interpolated via a spherical‐spline interpolation.

### 1.6 Error‐Related Negativity (ERN)

The ERN was measured as the mean amplitude from a cluster of frontal electrodes surrounding Fz (EGI electrodes 5, 10, 11, 12, 16, 18) for the first 100 ms following an incongruent error response. This Fz‐centered region of interest was chosen due to Fz having the numerically largest (most negative) ERN amplitude of the midline electrodes Fz, FCz, Cz, and Pz. The correct‐related negativity (CRN) was measured in the same way as the ERN except that it was measured from incongruent correct trials.

### 1.7 Data Analytic Strategy

Hypotheses were tested via a path model implemented in a structural equation modeling (SEM) framework. Predictors included maternal education, sex, the authoritarian, authoritative, and permissive subscales from the PSDQ, and relevant two‐ and three‐way interactions (see Table 2). Pubertal stage was also included as a covariate and was assessed via self‐report with the Pubertal Development Scale (Petersen et al. 1988). Prior to computing interaction terms, all predictors were mean‐centered. To better isolate the effects of predictors on the ERN versus on the CRN, both were included as outcomes within the same model. Given that our hypotheses only concerned the ERN, we only interpreted significant findings in the ERN and not the CRN. Analyses were conducted via a path model using full information maximum likelihood (FIML) estimation. Little's missing completely at random test (MCAR) suggested that data on all variables of interest were likely missing completely at random, χ^2^ (291)  =  22.562, *p* = 0.427. For information about missing data patterns (see Figure S1). The FIML estimator has been shown to provide unbiased estimates particularly when data are missing at random (Enders and Bandalos 2001). The model was tested in R, using the “lavaan” package (version 0.6‐17; Rosseel 2012). Significant interactions were probed using the “semTools” package in R (version 0.5‐6; Jorgenson et al. 2018). To probe the three‐way interaction estimate changes in ERN, the R function “probe3wayMC” was used, with parenting style as the predictor and both maternal education and sex as covariates.

**TABLE 2:** Predictors of ERN and CRN amplitude with covariates.

	Outcome					
	ERN			CRN		
Predictors	Estimate	Standard error	*p*	Estimate	Standard error	*p*
Sex	−0.694	0.472	0.142	0.259	0.255	0.309
ArP	−0.003	0.046	0.953	−0.036	0.032	0.260
AtP	−0.022	0.023	0.346	−0.027	0.017	0.110
PP	0.097	0.072	0.175	0.028	0.045	0.529
ME	0.159	0.436	0.715	−0.347	0.288	0.228
ME * ArP	0.258	0.077	**0.001**	−0.079	0.056	0.157
ME * AtP	0.024	0.033	0.464	−0.009	0.028	0.761
ME * PP	−0.248	0.076	**0.001**	−0.051	0.071	0.473
Sex * ArP	0.042	0.066	0.528	0.053	0.046	0.249
Sex * AtP	0.007	0.036	0.851	0.028	0.024	0.246
Sex * PP	−0.137	0.095	0.151	0.031	0.059	0.598
Sex * ME	0.215	0.591	0.717	0.886	0.381	**0.020**
ME * ArP * Sex	−0.292	0.093	**0.002**	0.092	0.072	0.201
ME * AtP * Sex	0.016	0.053	0.766	−0.044	0.041	0.280
ME * PP * Sex	0.402	0.101	**< 0.001**	0.046	0.084	0.586
Puberty Scale	−0.097	0.458	0.832	0.210	0.280	0.454

ArP = authoritarian parenting; AtP = authoritative parenting; CRN = correct‐related negativity; ERN = error‐related negativity; ME = maternal education; PP = permissive parenting.

* = interaction between variables bold values = statistically significant

## 2 Results

The relation between maternal education and ERN amplitude was assessed with a Pearson's correlation test. This test revealed no significant correlation (*r*(118) = 0.07, *p* > 0.05).

To test for potential interactions between maternal education and parenting style in predicting ERN amplitudes, a combined regression model was tested for each of the three parenting style subscales.

**FIGURE 1:**
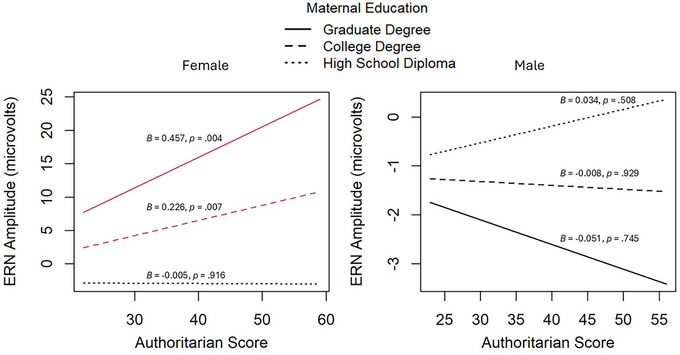
Three‐way interaction between maternal education, authoritarian parenting, and sex in predicting ERN amplitude. Note. Red lines indicate significant slopes (*p* < 0.05).

**FIGURE 2:**
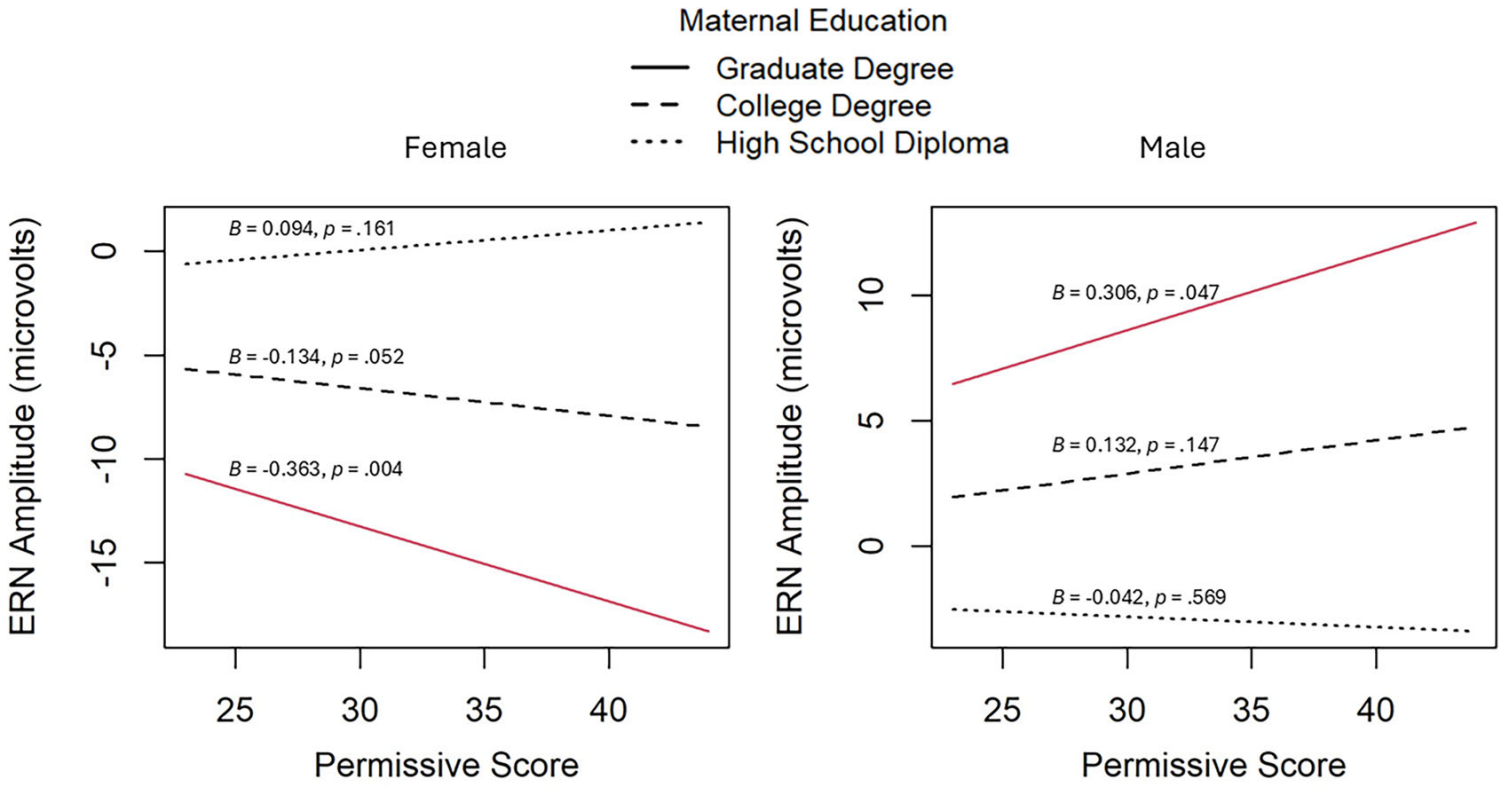
Three‐way interaction between maternal education, permissive parenting, and sex in predicting ERN amplitude. Note. Red lines indicate significant slopes (*p* < 0.05).

In the combined model, the three‐way interaction between authoritarian parenting, maternal education, and sex was significant (*p* = 0.002; Figure 1). Among female participants with higher levels of maternal education (i.e., college or postgraduate degree), greater authoritarian parenting was associated with smaller (less negative) ERNs. In female participants with lower levels of maternal education, increased authoritarian parenting did not significantly relate to ERN amplitude. Additionally, among males, authoritarian parenting scores did not significantly relate to ERN amplitudes at any level of maternal education. The three‐way interaction involving permissive parenting, maternal education, and sex, was also significant (*p* < 0.001; Figure 2). In females of higher levels of maternal education (i.e., postgraduate degree), as permissive parenting increases, the ERNs become larger (more negative). In females of lower levels of maternal education, permissive parenting was unrelated to ERN amplitude. For males with higher maternal education (i.e., postgraduate degree), greater permissive parenting was instead associated with smaller (less negative) ERNs. For males of lower SES levels, permissive parenting did not significantly relate to ERN amplitude.

## 3 Discussion

The present study examined the effects of SES and parenting behaviors on adolescents’ neural error monitoring. Based on previous work linking greater household income to larger ERN amplitudes (Perera‐W et al. 2021; Chong, Mirzadegan, and Meyer 2020), we expected individuals with higher SES to have larger ERNs. However, considering findings that maternal sensitivity may buffer the relation between SES and ERN amplitude (e.g., Brooker 2018), we also hypothesized that parenting style might moderate the SES‐ERN association. We found no direct effect of SES on ERN amplitude; however, we did find evidence that parenting style (specifically, authoritarian and permissive parenting) may moderate this association. In both cases, however, this moderation differed across males and females. For permissive parenting, simple slopes analyses revealed that among females at higher SES, more permissive parenting was associated with a larger (more negative) ERN. Conversely, among higher SES males, more permissive parenting was instead associated with a smaller (less negative) ERN. For authoritarian parenting, simple slopes analyses revealed that in females from medium to high SES backgrounds (maternal education attainment between college and postgraduate degree), higher authoritarian parenting was associated with smaller (more positive) ERN amplitudes. In males, at all SES levels, higher authoritarian parenting was not associated with any ERN amplitude changes.

Prior research suggests that permissive parenting does not reliably predict ERN amplitude (Chong, Mirzadegan, and Meyer 2020). The present findings suggest this is because the effect of permissive parenting on the ERN instead differs as a function of both the SES context and child sex. Existing work has found that high maternal permissive parenting is associated with heightened stress and anxiety for females but not for males (Barton and Kirtley 2012). Moreover, higher permissive parenting is associated with lower emotional regulation and control in children (Repetti, Taylor, and Seeman 2002). This finding, combined with the present findings, may suggest that heightened error sensitivity is one potential mechanism explaining the permissive‐ERN link, at least among females. A relative lack of emotional resources and parental support, which is commonly associated with permissive parenting, may lead to greater sensitivity to errors specifically among high SES females. Moreover, based on previous work in female adolescents, frequency of stressful life events during childhood was associated with more negative ERNs (Mehra, Hajcak, and Meyer 2022).

The lower ERN in males who experienced greater permissive parenting may be explained by previous findings that greater maternal permissive parenting is associated with greater impulsivity and risk‐taking in males (Patock‐Peckham et al. 2011). However, among females, greater maternal permissiveness was not associated with impulsivity. Research indicates that ERN amplitude is negatively correlated with risk‐taking and impulsivity in adolescent males and females (Taylor et al. 2018). Therefore, it may be that with greater maternal permissiveness, greater impulsivity develops in adolescent males, leading to decreased monitoring of errors, resulting in ERNs with smaller amplitudes.

Previous research suggests that in females, greater maternal authoritarian parenting is associated with an enhanced ERN amplitude (Banica, Sandre, and Weinberg 2019). It is hypothesized that punishing children's mistakes, a characteristic of authoritarian parenting (Kawamura, Frost, and Harmatz 2002; Robinson et al. 1995; Thompson, Hollis, and Dagger 2003), heightens children's error monitoring, manifesting as a larger ERN (Meyer and Gawlowska 2017; Riesel et al. 2012). Our findings add nuance to this past work. We found that the effect of authoritarian parenting differed as a function of SES and child sex. Specifically, at both medium and high SES, as maternal authoritarian parenting increased, ERN amplitude became smaller (more positive). Interestingly, research has indicated that in families of higher income, greater maternal authoritarian parenting levels are associated with greater risk‐taking in females but not males (Wood and Kennison 2017). It may be that the greater risk‐taking propensity developed in females of high SES who experience greater maternal authoritarian parenting is associated with decreased error monitoring, resulting in smaller (more positive) ERNs. Overall, our findings differ from our hypothesis that permissive and authoritarian parenting, along with low SES conditions, lead to increased (more negative) ERN amplitudes in females only. Instead, our findings suggest that more authoritarian parenting leads to decreased (more positive) ERN amplitudes at medium to high SES in females only. Additionally, our findings suggest permissive‐parenting‐related ERN differences for both males and females at high SES. Specifically, in high‐SES females, greater permissive parenting was associated with larger (more negative) ERNs. However, in high‐SES males, greater permissive parenting was instead associated with smaller (less negative) ERNs.

This study had several notable strengths and limitations. A key strength of this study was that it drew on multiple sources of information (e.g., parent report and electrophysiology) to test integrative hypotheses concerning the links among SES, parenting, sex, and cognitive control. Another important strength was the relatively large sample size, with most participants providing data for most of the measures. Moreover, the sex ratio among participants was approximately equal, with 46.39% of the sample being male. The relatively even distribution of male and female participants likely allowed sufficient statistical power to test interactions involving sex. A limitation is that although most of the PSDQ surveys were completed by the participants’ mothers, at least a small number were completed by the fathers, making it more difficult to attribute effects to a specific parent. Additionally, although the authoritarian and authoritative subscales of the PSDQ had acceptable internal consistencies, the permissive subscale's internal consistency was poor (Cronbach's alpha = 0.57)—likely due to the smaller number of items—limiting the interpretability of effects concerning this subscale. Another key limitation concerned the use of parental education as a proxy for SES. Although SES was a key component of this study and was included in most of the analyses, we had to rely entirely on the mother's reported highest level of education in the absence of any income data. Moreover, maternal education was measured on an ordinal scale such that high school diploma = 0, college degree = 1, and postgraduate degree = 2, possibly limiting the amount of variability on this measure. Lastly, the mean score on this measure was 1.2, meaning that the average participant mother had greater than a college degree, likely limiting the generalizability of the present findings.

Overall, the present study highlights (1) that parenting behaviors may modulate the impact of SES on cognitive control, and (2) the importance of considering sex differences when examining the interplay between SES, parenting, and cognitive control. Future work would benefit from including more comprehensive measures of SES (including family income and neighborhood characteristics), from testing the potentially unique contributions of paternal and maternal parenting behaviors, and from testing potential changes in the associations among SES, sex, and cognitive control across development.

## Conflicts of Interest

All authors report no biomedical financial interests or potential conflicts of interest.

## Supporting information



Figure S1. Visualization of missing data patterns. Note: Missing data are shown in red. ERN = error‐related negativity; CRN = correct‐related negativity.

Supporting information

## Data Availability

The data that support the findings of this study are openly available in NIMH Data Archive at https://nda.nih.gov/edit_collection.html?id=2538, reference number 2538.
