# Supplementary figures and images for: Sex Differences in the Associations Among Parenting, Socioeconomic Status, and Error Monitoring Among Adolescents
Source: Dev Psychobiol. 2025 Feb 11;67(2):e70023. doi: 10.1002/dev.70023 (PMC11814918; doi:10.1002/dev.70023)

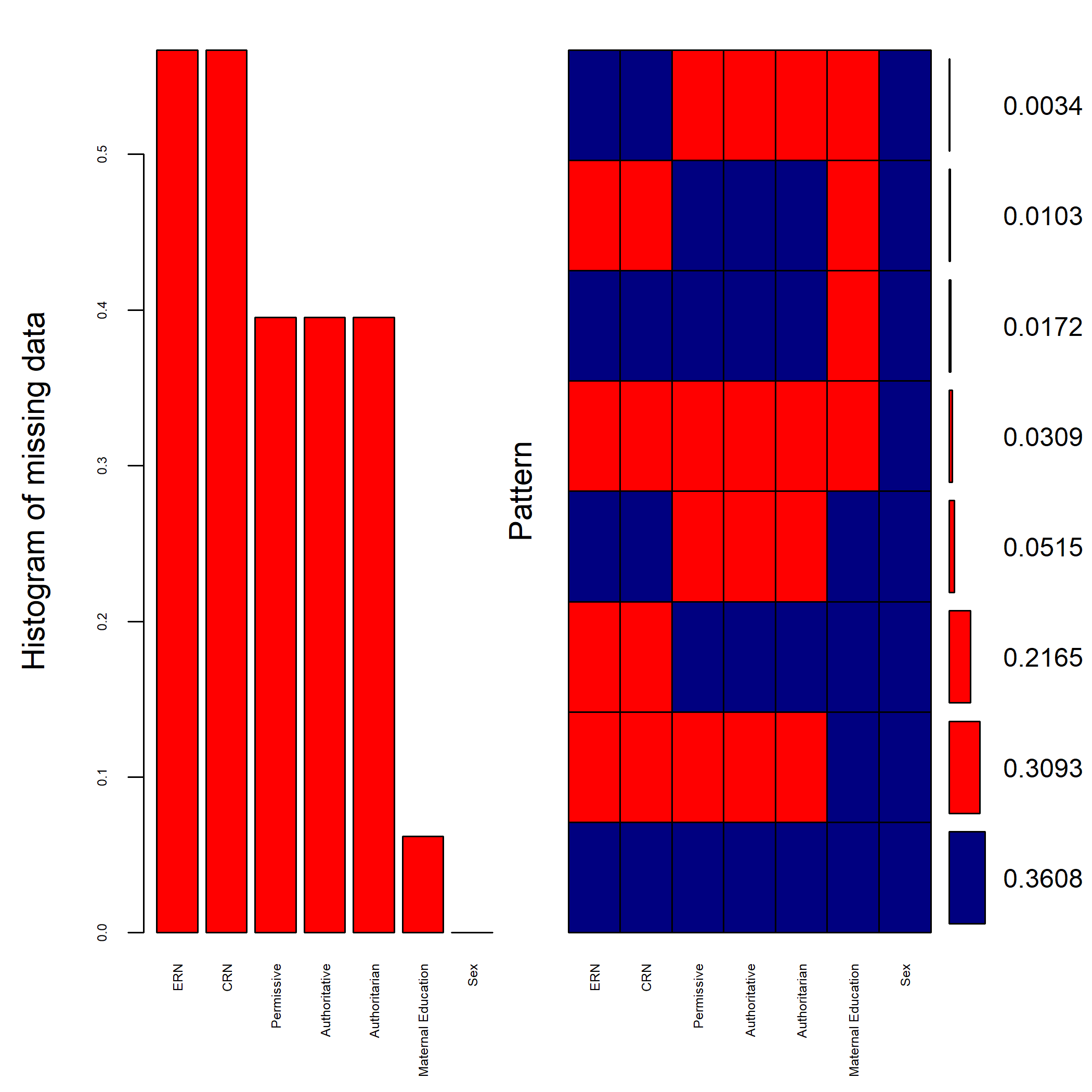

Supplement: Supplementary file 1 — Figure S1. Visualization of missing data patterns. Note: Missing data are shown in red. ERN = error‐related negativity; CRN = correct‐related negativity. [file DEV-67-e70023-s001.png]
