# Supplementary material for: Sex Differences in the Associations Among Parenting, Socioeconomic Status, and Error Monitoring Among Adolescents
Source: Dev Psychobiol. 2025 Feb 11;67(2):e70023. doi: 10.1002/dev.70023 (PMC11814918; doi:10.1002/dev.70023)
Supplement: Supplementary file 2 — Supporting information [file DEV-67-e70023-s002.docx]

**Supplement**

**Sex Differences in the Associations Among Parenting, Socio-economic Status, and Error Monitoring Among Adolescents**

*Figure S1. Visualization of Missing Data Patterns.*


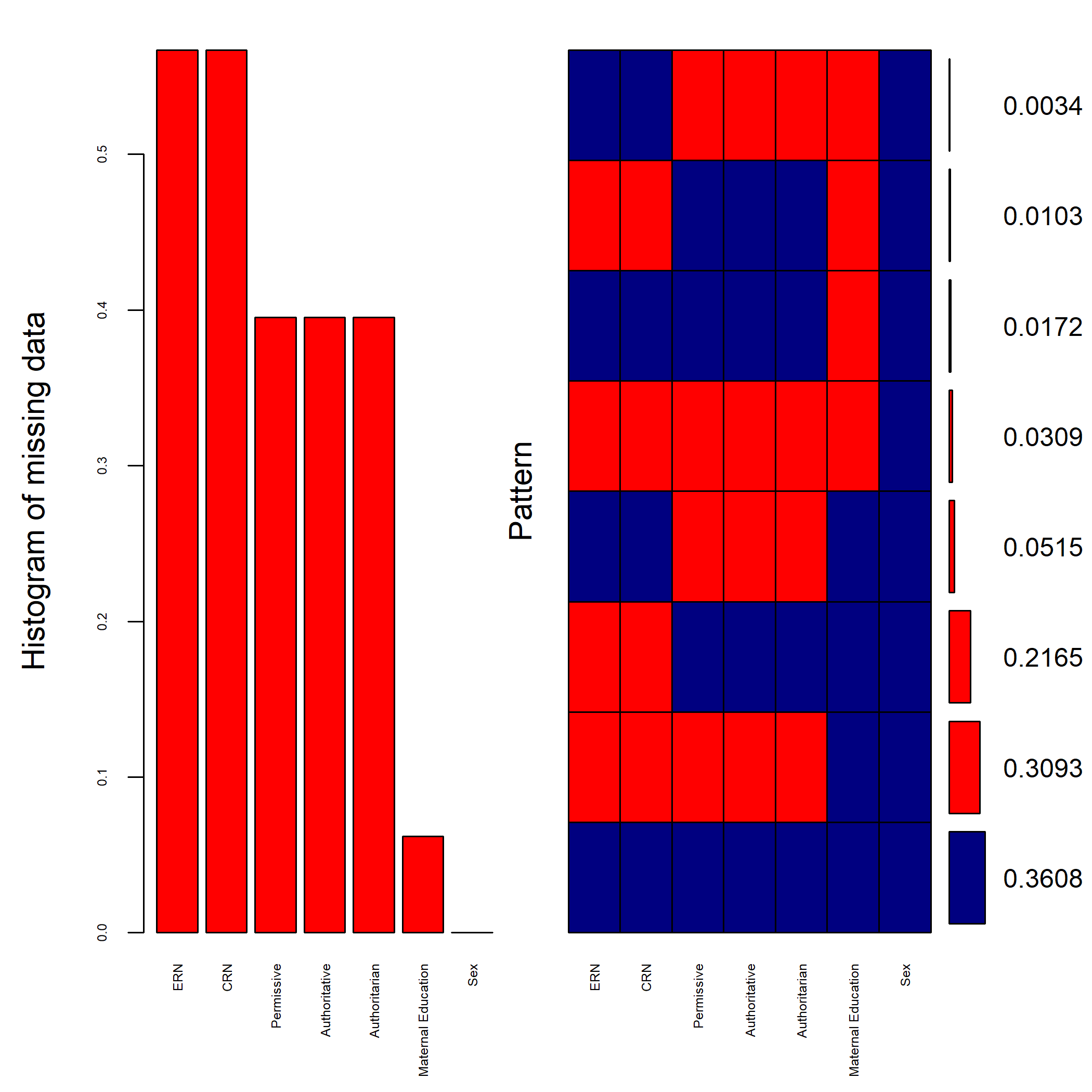


*Note:* Missing data are shown in red. ERN = error-related negativity. CRN = correct-related negativity.
